# Effects of Using Mechanical Brushes on the Productive Performance of Dairy Cows

**DOI:** 10.3390/vetsci11100481

**Published:** 2024-10-07

**Authors:** Hanbing Li, Ruixue Zhang, Haijing Li, Haojie Yuan, Ruihang Zhang, Hongyu Ren, Jinbang Xiao, Zuhui Li, Aihua Wang, Yaping Jin, Pengfei Lin

**Affiliations:** 1 Department of Clinical Veterinary Medicine, College of Veterinary Medicine, Northwest A&F University, Yangling 712100, China; lihanbing@nwafu.edu.cn (H.L.); zhangruixue@nwafu.edu.cn (R.Z.); lihaijing@nwafu.edu.cn (H.L.); 1476325831@nwafu.edu.cn (H.Y.); ZRH@nwafu.edu.cn (R.Z.); 2022055588@nwafu.edu.cn (H.R.); 15680830507@nwafu.edu.cn (J.X.); lizuhui123@nwafu.edu.cn (Z.L.); aihuawang1966@163.com (A.W.); 2 Key Laboratory of Animal Biotechnology, Ministry of Agriculture and Rural Affairs, Northwest A&F University, Yangling 712100, China; 3 Department of Preventive Veterinary Medicine, College of Veterinary Medicine, Northwest A&F University, Yangling 712100, China

**Keywords:** dairy cows, mechanical brush, productive performance, grooming behavior

## Abstract

**Simple Summary:**

A mechanical brush is currently used as a grooming tool to enhance the well-being of dairy cows and improve their overall environment. In the present study, we collected comprehensive data on the use of mechanical brushes by domestic dairy cows during the lactation, dry, and perinatal periods and the consequent effects on their lactation performance, health status, and reproductive performance. The findings of our study suggest that the frequency and duration of mechanical brush usage were significantly higher for brushes installed on the middle door than for brushes installed near the wall. Furthermore, the head was the preferred body part for grooming during the lactation and dry periods. Mechanical brush usage reduced postpartum stress in dairy cows, promoted blood circulation, improved the cleanliness of the body’s surface, enhanced milk production of lactating cows, and increased the progesterone concentration of pregnant dairy cows after artificial insemination (AI). Thus, the installation of mechanical brushes at appropriate positions in dairy farms can improve the production performance of dairy cows and enhance milk yield.

**Abstract:**

Intensive farming can reduce production costs and maximize animal production efficiency; however, it also causes many adverse effects on the welfare of dairy cows. A mechanical brush is an automated grooming device that promotes the grooming behavior of dairy cattle, thereby helping to alleviate stress. In the present study, we evaluated the effects of using mechanical brushes on the production performance of dairy cows by comprehensively analyzing their milk production, health status, and reproductive performance. The cows were assigned to 6 groups: 109 lactating dairy cows (brush treatment) and 105 controls (without brush treatment), 64 dry milk dairy cows (brush treatment) and 49 controls (without brush treatment), and 198 perinatal cows (brush treatment) and 65 controls (without brush treatment). We found an increasing trend in the daily utility time and usage frequency of mechanical brushes for each cow during the lactating period (7.73 ± 4.02 min/d and 2.90 ± 1.22 times/d, respectively), dry period (15.97 ± 14.16 min/d and 4.21 ± 2.91 times/d, respectively), and perinatal period (25.15 ± 19.05 min/d and 5.45 ± 3.83 times/d, respectively) (*p* < 0.01 and *p* < 0.05, respectively). The installation location of the mechanical brush significantly affected the frequency of its usage during the different periods. The head was the preferred body part for using the mechanical brush during the lactation and dry periods (59.32% and 44.54%, respectively), while the hip was the main preferred grooming part during the perinatal period (40.17%). Overall, the time, frequency, and preferred body part of dairy cows that used mechanical brushes varied across different physiological stages. Additionally, mechanical brush use in lactating and dry dairy cows significantly improved cleanliness of the body’s surface (*p* < 0.05) and enhanced milk production of lactating cows (*p* < 0.01), particularly for cows with four and five parities. Thus, the use of mechanical brushes could improve the production performance of dairy cows and enhance sustainability of large-scale farms.

## 1. Introduction

Following the increase in the scale of dairy farms globally, the dairy industry has become an important source of nutrition for consumers [1]. In recent years, consumers have become increasingly concerned about the welfare of dairy cows, as it is closely associated with milk quality, stable supply of raw milk, dairy-related materials, and economic benefits [2]. High-welfare dairy farming plays a crucial role in the sustainable development of dairy production [3].

In addition to feeding and resting, grooming is an important behavior for dairy cows. In a poor feeding environment, besides tongue licking and slapping the body surface with the tail for self-grooming, cows can rub their body surfaces only against metal gates, fences, and troughs; this behavior negatively affects their grooming efficiency and welfare [4]. Previous studies have shown that dairy cows receive adequate tactile stimulation through grooming by using a mechanical brush [5]. This approach meets the needs of dairy cows for hard-to-reach areas and helps alleviate their abnormal behavior [6,7]. DeVries [8] found that the utility time and frequency of grooming substantially increased when the mechanical brush was fixed in pens. Overall, the use of mechanical brushes is helpful in understanding social behavior in dairy cows and reducing stress caused by intensive management systems.

A mechanical brush is currently being used as a tool to meet the grooming needs of dairy cows and improve their overall environment [9,10]. This practice has gained immense popularity among dairy farmers worldwide. Mechanical brush application can more efficiently meet the grooming requirements of cows and enhance their milk production, particularly for cows in parity 2 [11]. In cows raised on farms, the use of mechanical brushes increased milk production by 8–17.6% [12,13]. There is, however, limited research on the effect of mechanical brush on the 305-day milk production, health, and reproductive performance of dairy cows [14,15]. Currently, there are no studies that have concurrently analyzed the mechanical brush data of cows across different physiological stages (lactation, dry, and perinatal). There is also a lack of comprehensive data regarding this topic in China as well as a scarcity of studies evaluating the overall effect of using mechanical brushes on dairy cows.

The present study aimed to collect comprehensive data on the use of mechanical brushes by domestic dairy cows during the lactation, dry, and perinatal periods and the consequent effects on their lactation performance, health status, and reproductive performance. We hypothesized that there are differences in the frequency of mechanical brush use in dairy cows in different physiological stages, with the highest frequency of mechanical brush use in perinatal cows. Furthermore, it was anticipated that the implementation of mechanical brushes would enhance the production performance of cows, particularly in terms of milk production.

## 2. Materials and Methods

From April to October 2022, we conducted an experiment in a commercial dairy farm in northwest China. This study was approved by the Committee for the Ethics on Animal Care and Experiments, Northwest A&F University.

### 2.1. Animals and Housing

For this experiment, early lactating dairy cows (<40 days postpartum), dry dairy cows (220–250 days gestation), and perinatal dairy cows (>250 days gestation without delivery) were enrolled in the study and randomly housed in pens with mechanical brushes or control pens without a mechanical brush. The commercial ranch management software, Yi Muyun (Yimu Technology Co., Ltd., Beijing, China), was used daily for the comprehensive management of dairy cows. This included overseeing breeding programs, disease detection, and milk volume recording.

**Figure 1:**
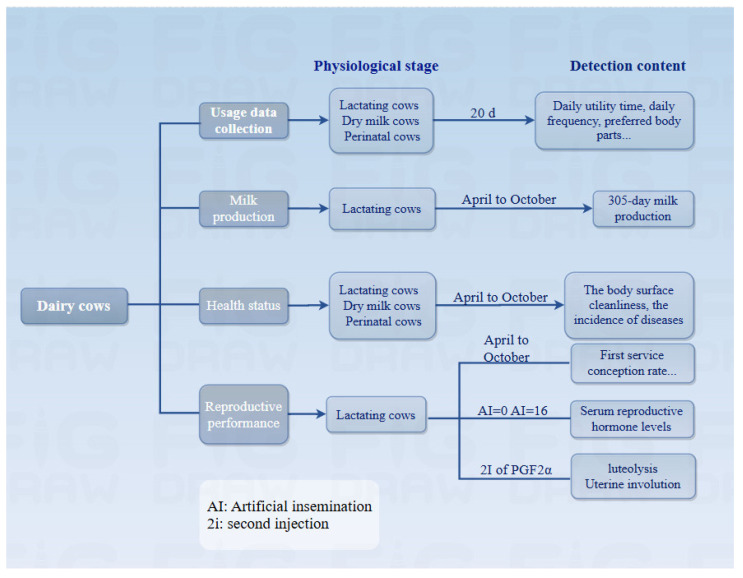
Flowchart of the experiment.

The total number of Holstein dairy cows in the herd was over 2800. Among these cows, 48.1%, 14.9%, and 14.9% were lactating, dry, and perinatal cows, respectively. The experimental animals had an average 305-day milk production of 10,955 kg over a period, with an average of 3.27 parities. All dairy cows were housed in free pens, fed a total mixed ration, and given free access to feed and water. All pens were equipped with an exercise yard, with free access. Lactating cows prioritized feeding and water intake after milking, specifically during 6:00–7:00 AM, 2:00–3:00 PM, and 10:00–11:00 PM. Dry and perinatal cows prioritized feeding and water intake during 6:00–7:00 AM and 2:00–3:00 PM. During the test, the temperature, relative humidity, and temperature-humidity index (THI) were in the range of 0–39.5 °C, 17–99%, and 34–90, respectively. Animal facilities, herd management, and herd structure remained unchanged during the test. The test procedures are shown below (Figure 1).

### 2.2. Mechanical Brush Installation and Usage Data Collection

**Figure 2:**
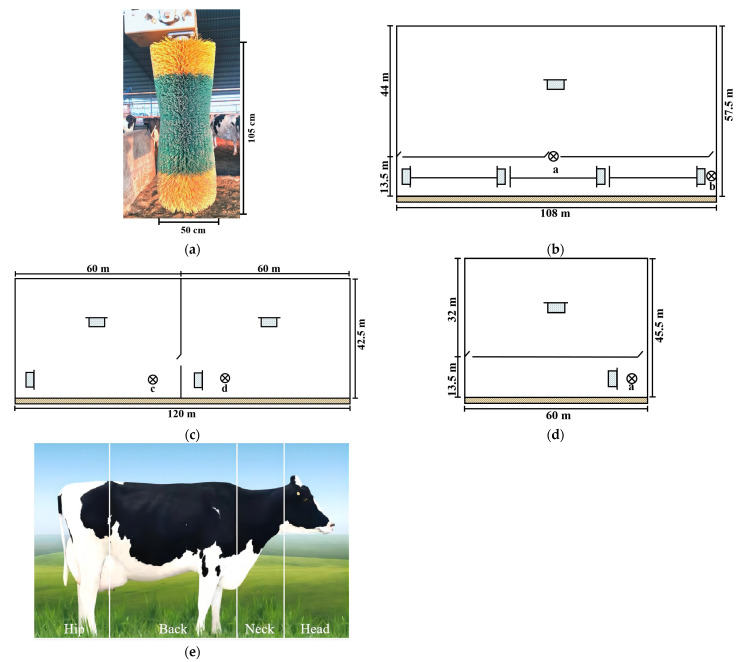
Experimental area, illustration of a mechanical brush, and the preferred body parts used by the cows. (**a**) Lactation livestock house size and installation locations of mechanical brushes. (**b**) Perinatal livestock house size and installation locations of mechanical brushes. (**c**) Dry milk livestock house size and installation locations of mechanical brushes. (**d**) Mechanical brush and its installation dimension. (**e**) The preferred body parts used by the cows. Note: 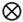 Mechanical brush; 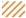 Feeding trough; 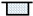 Water trough; (a, b, c, d) represent the installation positions of different mechanical brushes.

Electrical mechanical brushes (diameter: 32 cm, length: 105 cm, Qingdao Wenfeng Industrial Co., Ltd., Qingdao, China; Figure 2a) were hung in the pen specifically designed to house 60 cows [9]. These were two-headed brushes with hard nylon bristles and soft green plastic bristles in the middle part. Five brushes were installed at 0.8 m above the ground. Two brushes were installed in the lactation enclosure: one on the middle door and one near the wall (Figure 2b). The remaining two brushes were installed in the perinatal enclosure: one near the water trough and the other distant from the water trough (Figure 2c). Locations a and b indicate that mechanical brushes were installed on the middle door and near the wall of the lactation livestock house, respectively. Additionally, locations c and d indicate that mechanical brushes were installed distant from the water trough and near the water trough of the perinatal livestock house, respectively. Another brush was installed near the water trough in the dry milk enclosure (Figure 2d).

Prior to the experiment, all cows underwent a one-week adaptation period. Subsequently, video cameras (DS-2CD3T47EDWDV3-L, Hikvision, Hangzhou, China) were used to collect data by identifying ear tags from video recordings. In this experiment, the mechanical brush was rotated with a tilt angle of less than 15 degrees. A duration of more than 30 s for each cow was considered for data collection. The mechanical brush use data were recorded from dairy cows during the lactation, dry milk, and perinatal periods for 20 days. According to the preferred body parts used by the cows for grooming with the mechanical brush, data were collected from the following four body parts: head, neck, back, and hip (Figure 2e) [8].

The preferred body parts were determined based on the duration of mechanical brush use and number of body parts. The body part used for a longer duration as compared to the other body parts was considered the preferred body part. If different body parts were used for a similar length of time, they were not considered the preferred body parts. The frequency of using a specific body part indicated its preference for grooming. For example, if a particular body part of dairy cows were used for more than 50% of the total time, it was considered the preferred body part. If two body parts were used, each part was considered to account for more than 40% of the total time of mechanical brush use. If three body parts were used, each part was considered to account for more than 30% of the total time of mechanical brush use.

For comprehensive statistical analysis of the usage of mechanical brushes, the following parameters were considered based on the ear tags of the corresponding cows: daily utility time, daily frequency, daily peak period, number of cows, usage rate, and preferred body parts. The frequency and duration of mechanical brush use by cows at different physiological stages were compared, with the data from lactating and perinatal cows selected as the average value of mechanical brushes installed in pens.

The total recorded time per day of mechanical brush use was calculated in hours per day (h/d), while the usage time in different time periods was calculated in minutes per hour (min/h). The average utility time was used to calculate the usage time per day in minutes (min/d). The frequency per day was measured in times per day, while the frequency in different time periods was counted in times per hour (times/h). The group of animals in this herd remained unchanged during the data collection process. The data for cows with low frequency or no daily use of mechanical brush were excluded.

### 2.3. Collection of Data Related to Lactation, Disease, and Reproduction of Dairy Cows

By using Yi Muyun management software (2.0.0), data on the 305-day milk production per lactation cow were collected from both control and experimental group dairy cows. Additionally, data on disease type, incidence time, cure rate, first service conception rate, days to the first service, number of services per conception, number of days open, and abortion rate of dairy cows were collected from Yi Muyun management software. During the experiment, the milk production of lactating dairy cows was recorded from cows with two or more parities; cows with similar milk production in the last parity were chosen for accurately assessing the effects of mechanical brush usage. Furthermore, the breeding data of lactating cows in the same mating month were collected.

### 2.4. Assessment of Cow Body’s Surface Cleanliness

Body cleanliness score was defined according to the condition of the skin surface of dairy cows, including the udder skin. The detailed scoring criteria for determining cleanliness based on the surface dirt area were as follows [16]: 1 (body surface area free of dirt), 2 (1–10% of the body surface area covered with dirt), 3 (10–30% of the body surface area covered with dirt), and 4 (> 30% of the body surface area covered with dirt). Scores 1 and 2 indicated good hygiene of the body surface, while scores 3 and 4 indicated poor hygiene. During the trial, the assessments were conducted once a month at 3:00 PM (based on previous animal experimentation and ethics protocol).

### 2.5. Experimental Groups and Control Groups

All cows were assigned to 6 groups based on the physiological stage and installation/no installation of a mechanical brush: 109 lactating dairy cows and 105 controls, 64 dry milk dairy cows and 49 controls, and 198 perinatal cows and 65 controls.

B-ultrasound examination for detecting uterine involution and luteolysis was performed in 30 and 32 lactating cows from the control and experimental groups, respectively, and serum estradiol and progesterone concentrations were measured in 30 and 56 cows from the control and experimental groups, respectively.

### 2.6. Breeding Program and Detection of Uterine Involution and Luteolysis

All cows received Presynch–Ovsynch treatment [17]. If any signal of estrus were observed during program execution, the above-mentioned procedure was terminated, and insemination was performed in the dairy cows after 8 to 12 h. If no signal of estrus were observed, the procedure was strictly followed, and the cows were included in the timed artificial insemination (TAI) protocol after the procedure [18].

To further evaluate the effect of mechanical brush on the reproductive performance of dairy cows, uterine involution and luteolysis in postpartum dairy cows were detected using a B-mode veterinary ultrasound scanner (7.5 MHz, Easi-Scan, BCF Technology, Rochester, MN, USA) during the use of mechanical brushes at the second injection of PGF_2α_ in the pre-synchronization program. Ultrasound examination was conducted on dairy cows in the postpartum stage (between 55 and 60 days), and the reference standard was consistent with a previous study [19].

### 2.7. Detection of Serum Estradiol and Progesterone Concentrations by ELISA

To minimize the risk of errors, a consistent group of cows that had undergone AI were chosen to undergo coccygeal blood vessel puncture following their morning feeding. The blood samples were centrifuged at 3000 rpm for 15 min at 25 °C to obtain serum, which was frozen for further analysis. Blood samples were collected on days 0 and 16 (AI = 0) to assess progesterone and estradiol concentrations.

Progesterone concentration was determined using a kit in accordance with the manufacturer’s instructions (Jiangsu Meimian Industrial Co., Ltd., Jiangsu, China). The absorbance value was measured using an ELISA reader (Spark, Tecan, Switzerland) at 450 nm wavelength. Standard curves were plotted using known concentrations of standard samples, and the concentrations of the unknown samples were calculated based on the standard curves. The minimum detectable concentration of progesterone was 0.31 ng/mL, with intraplate and interplate coefficients of variation below 10%.

Estradiol concentration was determined using a kit in accordance with the manufacturer’s instructions (Nanjing Jiancheng, Nanjing, China). The absorbance value was measured using an ELISA reader (Spark, Tecan, Switzerland) at 450 nm wavelength. Standard curves were plotted using known concentrations of standard samples, and the concentrations of the unknown samples were calculated based on the standard curves. The minimum detectable concentration of progesterone was 3 pg/mL, with intraplate coefficients of variation below 10% and interplate coefficients of variation below 12%.

### 2.8. Statistical Analysis

All data were collected and organized using Microsoft Excel. Statistical analysis was performed using GraphPad Prism 9 software. The experimental results were expressed as mean ± standard deviation (SD). The Chi-square test and Fisher’s exact test were used to analyze the data for disease incidence rates, uterine involution rate, first service conception rate, and abortion rate of lactating cows. The Mann–Whitney *U* test was used to analyze the data for the preferred body parts. Additionally, estradiol and progesterone concentrations and the utility time and frequency of usage of mechanical brushes were analyzed by one-way ANOVA and *t*-test. The t-test was used for the statistical analysis of 305-day milk production, number of services per conception, number of days open, and body surface cleanliness. *p* < 0.05 indicated a significant difference.

## 3. Results

### 3.1. Analysis of the Use of Mechanical Brushes by Dairy Cows at Different Physiological Stages

**Figure 3:**
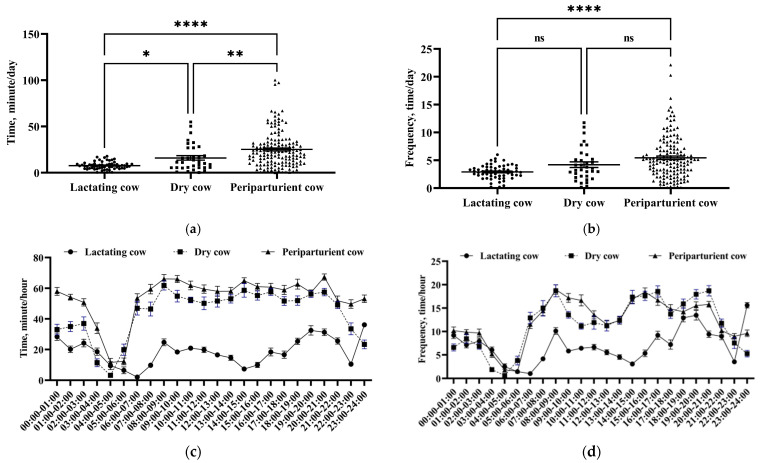
The daily utility time and frequency of usage of mechanical brushes by cows at different physiological stages. The daily utility time (**a**) and daily frequency of usage (**b**) of mechanical brushes for each cow. The daily utility time (**c**) and daily frequency of usage (**d**) of mechanical brushes at different time periods. The values in the chart are expressed as average ± standard error. ns indicates no significant change, * (*p* < 0.05), ** (*p* < 0.01) and **** (*p* < 0.0001) indicate significant differences.

Figure 3a shows the average daily utility time of mechanical brushes by each cow in the lactation, dry, and perinatal periods. Dairy cows in the perinatal period (25.15 ± 19.05 min/d; *n* = 160) showed a significantly longer duration of mechanical brush use than those in the lactation period (7.73 ± 4.02 min/d; *n* = 59) and the dry period (15.97 ± 14.16 min/d; *n* = 34) (*p* < 0.01). Additionally, the mechanical brush utility time was significantly longer for cows in the dry period than for cows in the lactation period (*p* < 0.05).

The average daily frequency of mechanical brush use by each cow exhibited similar trends for lactating, dry, and perinatal cows (Figure 3b). The average frequency of mechanical brush usage per cow was significantly higher in the perinatal period (5.45 ± 3.83 times/d) than in the lactation period (2.90 ± 1.22 times/d) (*p* < 0.01). However, no significant difference was noted in the average daily frequency of mechanical brush usage per cow between lactating cows and dry cows (*p* = 0.158) and between perinatal cows and dry cows (4.21 ± 2.91 times/d) (*p* = 0.115).

Figure 3c shows the average time of mechanical brush use by cows for different time periods during the lactation, dry, and perinatal periods. Cows in the lactation period showed significantly lower usage of mechanical brushes at different time periods as compared to cows in the dry and perinatal periods, particularly after 6:00 AM; however, no significant differences in brush usage were observed between the dry and perinatal periods. The lowest usage of mechanical brushes was recorded between 3:00 and 6:00 AM. This finding indicated differences in the utility time of mechanical brushes at different stages and different time periods. The average frequency and time of mechanical brush usage by dairy cows exhibited similar trends at different time periods in the lactation, dry, and perinatal periods (Figure 3d).

**Figure 4:**
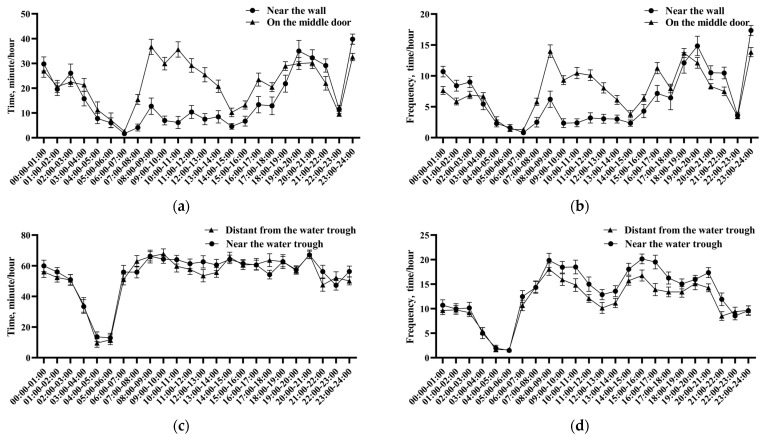
Effects of different installation locations on the duration and frequency of mechanical brush use in different physiological stages of dairy cows. (**a**) Utility time of mechanical brushes at different time periods in lactating dairy cows. (**b**) Frequency of usage of mechanical brushes at different time periods in lactating dairy cows. (**c**) Utility time of mechanical brushes at different time periods in perinatal dairy cows. (**d**) Frequency of usage of mechanical brushes at different time periods in perinatal dairy cows. The values in the chart are expressed as average ± standard error.

Figure 4a,b shows the time and frequency of mechanical brushes used by lactation cows at different time periods. The frequency of usage of the mechanical brush installed in the lactation livestock house by cows gradually increased after milking and feeding, particularly within 2 h after feeding. Moreover, significant differences were noted in the usage of the mechanical brush installed near the wall and that installed on the middle door at time intervals between 7:00 AM and 2:00 PM. The average utility time and frequency of usage of the mechanical brush installed on the middle door at different time periods were significantly higher than that installed near the wall (*p* < 0.01 and *p* < 0.05, respectively).

Figure 4c,d shows the time and frequency of mechanical brushes used by perinatal cows at different time periods. There was no fixed time for the feeding of dairy cows. The frequency and time of usage of mechanical brushes were the lowest in the early morning, particularly from 3:00 AM to 6:00 AM. Overall, perinatal cows used mechanical brushes for a longer duration as compared to lactation cows (*p* < 0.01). Additionally, the utility time of mechanical brushes for perinatal cows showed no significant differences at different time periods. However, perinatal cows showed a significantly higher frequency of using mechanical brushes near the water trough as compared to that distant from the water trough (*p* < 0.01).

**Figure 5:**
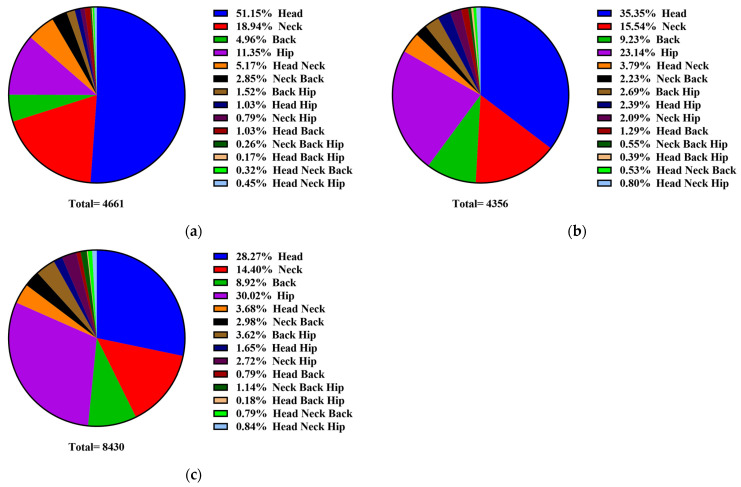
The preferred body parts used by the cows for grooming with mechanical brushes at different stages. (**a**) Lactating dairy cows. (**b**) Dry dairy cows. (**c**) Perinatal dairy cows.

Figure 5 shows the statistical results of the preferred body parts used by the cows for grooming with mechanical brushes at different stages. During lactation, the main preferred body parts of the cows for grooming were the head (51.15%), neck (18.94%), and hip (11.35%), and the remaining body parts accounted for a smaller percentage (Figure 4a). For dry cows, the main preferred body parts for grooming were the head (35.35%), hip (23.14%), and neck (15.54%). In perinatal cows, the main preferred body parts for grooming were the hip (30.02%), head (28.27%), and neck (14.40%). Overall, when using mechanical brushes, the hip and head tended to be the main preferred body parts of dairy cows at different lactation stages.

### 3.2. Effect of Using Mechanical Brushes on Milk Production in Dairy Cows

Previous studies have shown that second parity cows have a more significant increase in milk production after using mechanical brushes [11]. Table 1 shows the total 305-day milk production of dairy cows with different parities. Compared to the control group, the total 305-day milk production of second parity, third parity, fourth parity, fifth parity, and the whole group (all parity cows) increased by 927.97, 587.81, 2299.1, 1029.48, and 983.74 kg/cow, respectively.

Milk production showed no significant difference between the experimental and control groups for lactating cows in the second and third parities. However, a significant difference in milk production was observed for lactating cows in the fourth and fifth parities (*p* < 0.05).

### 3.3. Effect of Using Mechanical Brushes on the Health Status of Cows

**Figure 6:**
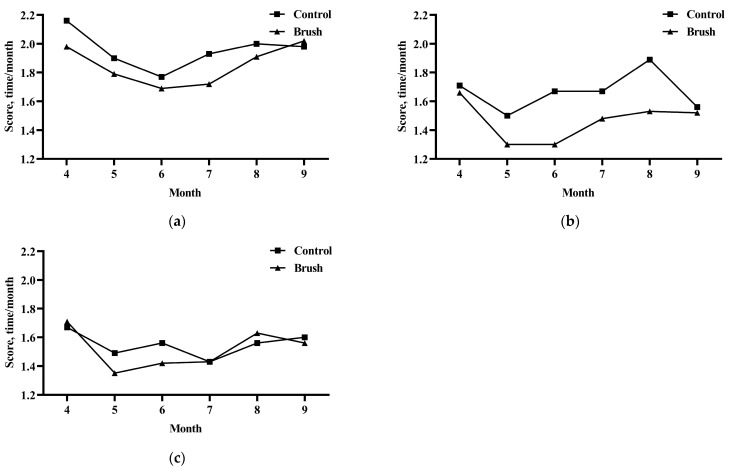
Effect of using mechanical brushes on the body surface cleanliness of dairy cows at different physiological stages. (**a**) Lactating dairy cows. (**b**) Dry dairy cows. (**c**) Perinatal dairy cows. The values in the chart are expressed as average.

As shown in Figure 6, the usage of mechanical brushes apparently enhanced the body surface cleanliness of dairy cows in the experimental group during the lactation and dry periods, as compared to that of cows in the control group (Figure 6a,b) (*p* < 0.05). However, no significant impact was observed on the body surface cleanliness of perinatal cows (Figure 6c). Moreover, the overall cleanliness of dairy cows was better during the dry and perinatal periods than during the lactation period. The farm also had a good sanitary environment, and mechanical brush usage improved the cleanliness and hygiene of dairy cows to a certain extent.

**Figure 7:**
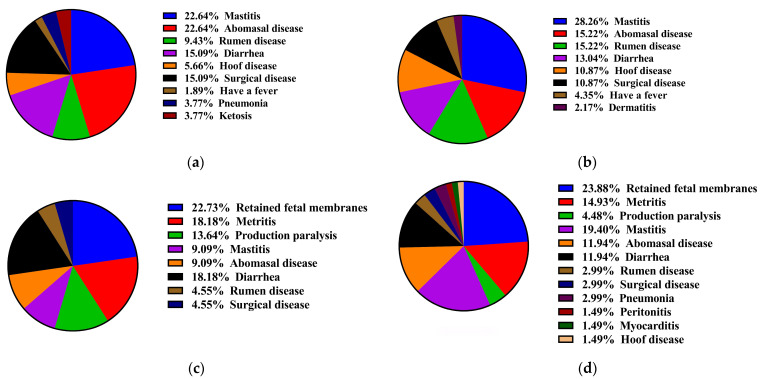
The incidence of diseases in dairy cows during the trial period. (**a**) Control group of lactating dairy cows. (**b**) Experimental group of lactating dairy cows. (**c**) Control group of perinatal dairy cows. (**d**) Experimental group of perinatal dairy cows.

Figure 7a,b shows the incidence of diseases in lactating dairy cows during the trial period. Of the 109 dairy cows in the experimental group, 38 cows showed sickness, resulting in a cumulative incidence of 46 times, and 7 of the 38 cows had multiple illness episodes. Therefore, the overall incidence rate in the experimental group was 34.86%. In the control group, 38 of 105 cows were sick with a cumulative incidence of 53 times, and 13 of the 38 cows had multiple illness episodes. The total incidence rate was 34.29%. Interestingly, the cows were healthy during the dry period. Figure 7c,d shows the incidence of diseases in perinatal cows (30 days postpartum). Of the 198 cows in the experimental group, 57 cows were sick, resulting in a cumulative incidence of 67 times, and 10 of the 57 cows had multiple illness episodes during the experimental period, resulting in a total incidence rate of 28.79%. In the control group, 17 of 65 cows were sick, with a cumulative incidence of 22 times, and 4 of these 17 cows had multiple illness episodes. The total incidence rate was 26.15%. Overall, mechanical brush usage had no significant effect on the disease incidence rate and types of diseases in dairy cows.

### 3.4. Effect of Mechanical Brush on the Reproductive Performance of Dairy Cows

To determine the effect of mechanical brush usage on uterine involution and estrus synchronization, the two groups of cows were examined by B-ultrasound three days after the second injection of PGF_2α_. As shown in Table 2, the experimental group exhibited a higher rate of uterine involution (70%) and luteolysis dissolution (100%) than the control group (55% and 95%). Moreover, the overall abortion rate was lower in the experimental group (10.94%) than in the control group (14.28%). Overall, the experimental and control groups showed no significant differences in various reproductive indicators.

**Figure 8:**
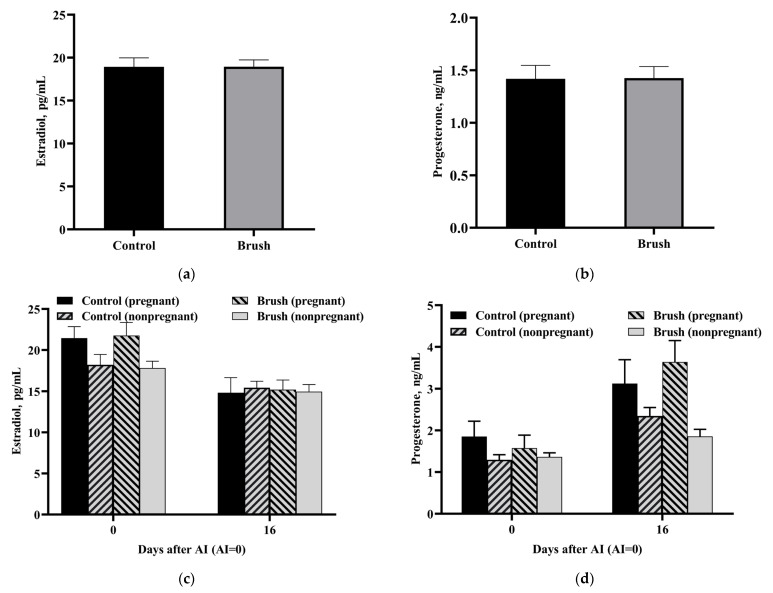
Changes in serum reproductive hormone levels in lactating dairy cows. (**a**) Estradiol levels in dairy cows on the day of AI. (**b**) Progesterone levels in dairy cows on the day of AI. (**c**) Estradiol levels in pregnant and nonpregnant dairy cows. (**d**) Progesterone levels in pregnant and nonpregnant dairy cows. The values in the chart are expressed as average ± standard error.

Figure 8a,b shows changes in the levels of reproductive hormones in the control and experimental groups on the day of the first AI. The experimental and control groups showed no significant changes in the concentrations of estradiol and progesterone.

To further investigate the effect of mechanical brush usage on the levels of reproductive hormones in pregnant and nonpregnant cows, each group was further subdivided into pregnant and nonpregnant groups (Figure 8c,d). In pregnant and nonpregnant cows, the estradiol and progesterone levels showed no significant difference between the experimental and control groups on the day of the first AI and on day 16 after the first AI. However, in the experimental group, the estradiol concentration of pregnant cows was significantly higher than that of nonpregnant cows on the day of the first AI (*p* < 0.05), while pregnant and nonpregnant cows in the control group showed no significant difference in the estradiol concentration. Interestingly, after AI, the average progesterone level of pregnant cows was higher in the experimental group than in the control group. However, the nonpregnant cows of the experimental group showed an apparent decrease in progesterone levels as compared to those of the control group.

Overall, compared to nonpregnant cows, the pregnant cows showed a significant increase in the estradiol level on the day of estrus and a higher level of progesterone on day 16 after the first AI; moreover, cows in the experimental group exhibited more significant changes in hormone levels than those in the control group.

## 4. Discussion

Recent studies carried out on weaned calves [20,21,22], lactating dairy cows [8,9,23], and perinatal dairy cows [24] have revealed that the fundamental motivations influencing the frequency and duration of mechanical brush utilization are intricately interwoven with numerous factors, such as ambient temperature, ambient noise levels, and accessibility of resources. Mandel [25] and Foris [9] demonstrated that dairy cows used mechanical brushes more frequently when these brushes were installed near water and feed troughs. Conversely, environments with high temperature and high humidity and the period leading up to AI had a negative effect on the usage of mechanical brushes in pens. In the present study, we observed that cows preferred to use mechanical brushes mounted on the middle door and those closer to the sink. This difference might be related to environmental noise, water requirements, and energy allocation. In a previous study, dominant cows used mechanical brushes (particularly during the peak feeding period) more frequently for an average of 270 ± 140 min/week, while subordinate cows used them for 114 ± 49 min/week; moreover, dominant cows used the brushes for 1.5-fold longer than subordinate cows [26]. Furthermore, certain cows exhibited increased access to the mechanical brush because of displaying aggressive behavior toward their fellow cows. Additionally, weaned calves used mechanical brushes for a longer duration when there were more brushes installed in the pens [27]. Our study showed that the duration and frequency of usage of mechanical brushes were very high in dry milk and perinatal cows. Thus, it may be necessary to enhance the grooming facilities in dry and perinatal pens by increasing the number of mechanical brushes. This adjustment would help ensure that the grooming needs of cows are adequately met, potentially by reducing the ratio of cows to mechanical brushes, for example, to 1:40. In conclusion, it is important to consider human activities, external sounds, environmental resources, cow numbers, and physiological stages when installing mechanical brushes to reduce group competition, meet grooming requirements, and decrease energy consumption as much as possible.

Previous findings suggest that dairy cows allocate a significant amount of their grooming time to tend to their head and neck regions during grooming activities [6,8,22,28,29]. In the present study, we observed that the head was the preferred body part for grooming during the lactation and dry periods; this finding also agreed with previous studies. A noteworthy finding is that the proportion of head grooming during the lactation, dry, and perinatal periods gradually decreased with the increase in lactation, while that of back and hip grooming gradually increased. This difference might be because of physical changes in the cow’s body as well as the increase in the abdominal circumference and abdominal pressure in cows during late pregnancy. Therefore, grooming the back and hips can help alleviate pressure and discomfort. As shown previously, mutual grooming behavior and mechanical brush usage mainly occurred in the afternoon, while other behaviors, such as tongue rolling and bar licking, were primarily performed during the midday [28]. In the present study, we observed that the dairy cows predominantly engaged in sleep behaviors from 3:00 AM to 6:00 AM with less frequent utilization of mechanical brushes. The frequency of mechanical brush usage was notably more frequent among dry and perinatal cows, with lactating cows showing the highest frequency of use at 2 h after feeding. It is worth noting that the frequency of mechanical brush usage during the lactation period was largely influenced by the month and changes in temperature. These factors contributed to a decrease in mechanical brush usage during lactation. Therefore, heat and cold stress should be considered for lactating dairy cows to avoid affecting their daily behavior.

DeVries [8] found that the usage of mechanical brushes can significantly increase the average milk production of dairy cows from 41.0 ± 2.6 to 47.2 ± 0.7 kg/d. Similarly, Ynte H [11] discovered that mechanical brushes significantly increased daily milk production by 3.5% (1 kg/d) for cows with two parities, while there was no significant difference between cows with one parity and those with greater than two parities. Chinese researchers have reported that the annual milk production of commercial dairy farms with mechanical brushes in Beijing suburbs was 8.3% higher than that of dairy farms without mechanical brushes [13], and the difference was 17.6% for dairy farms located in the southwest region [12]. Our present study supported these findings and demonstrated that mechanical brush usage can significantly increase milk production, particularly for cows with four and five parities. Therefore, the installation of a mechanical brush in early lactation dairy will improve the economic benefits of large-scale farms.

As reported previously, mechanical brush usage was beneficial in promoting blood circulation and milk production and for cleaning the body surface of dairy cows [30]. In the present study, the installed mechanical brushes significantly improved the body surface cleanliness of dairy cows during the lactation and dry periods; however, these brushes had no significant effect on body surface cleanliness during the perinatal period. The reason for this difference might be that the cows are relatively clean during the perinatal period. Therefore, the cleaning strength of the installed mechanical brushes was influenced by the cleanliness of the cow itself.

The frequency of using mechanical brushes was low when dairy cows had poor health [14,15]. Mechanical brush usage in barns can decrease the incidence of clinical mastitis in cows with two or more parities [11]. However, our present study showed no significant difference in mechanical brush usage between healthy and diseased dairy cows; this finding was inconsistent with previous studies. Another study demonstrated that the incidence of mastitis in dairy cows is associated with the season, with a significant increase in the incidence in hot and humid environments [31]. Therefore, we speculate that environmental factors may play a critical role in the incidence of clinical mastitis. The experimental period for this study was from April to September. The hot and humid environments during this time may have also influenced the incidence of dairy cow diseases, potentially explaining why there was no significant difference in disease incidence between the experimental group and the control group. Overall, we found that mechanical brushes can improve body surface cleanliness but do not significantly affect disease incidence.

Modern large-scale farms use simultaneous estrus and ovulation procedures to enhance work efficiency and improve the conception rate of cows; among these procedures, the Presynch–Ovsynch protocol is one of the most widely used programs for the first postnatal AI service [32]. Hormonal regulation, particularly progesterone and estradiol, plays a pivotal role in reproductive success [33,34]. Previous studies have shown that a low concentration of progesterone adversely affects embryo implantation and late embryonic development, leading to an increase in embryo abortion and a decrease in the reproductive performance of dairy cows [35,36]. In the present study, compared to the control group, the progesterone level in pregnant cows from the experimental group was higher post-AI. However, because of constraints in the research, the impact of mechanical brushes on progesterone levels in cows remains uncertain. The potential implications of this finding on the reproductive capacity of dairy cows warrant further investigations.

## 5. Conclusions

The results of the present study indicate that the time and frequency of using mechanical brushes exhibited an upward trend in lactating, dry, and perinatal cows, with differences in the preferred body parts for mechanical brush usage and a greater frequency of using brushes installed near the water trough and on the middle door of the livestock house. Although mechanical brushes can improve the milk production and body surface cleanliness of dairy cows, there is no apparent change in the morbidity rate and reproductive performance of dairy cows. These findings promote the application of mechanical brushes in large-scale dairy farming.

## Figures and Tables

**Table 1:** Total 305-Day Milk Production of Dairy Cows with Different Parities.

Parity	Brush		Control		*p*-Value
	Mean ± SD	N	Mean ± SD	N	
Second parity (kg)	12,343.88 ± 2329.79	8	11,415.91 ± 954.52	11	0.276
Third parity (kg)	12,752.10 ± 1587.09	39	12,164.29 ± 1754.10	35	0.140
Fourth parity (kg)	13,819.67 ± 1520.15 ^a^	9	11,520.57 ± 1976.49 ^b^	7	0.028
Fifth parity (kg)	13,992.29 ± 2155.13 ^a^	35	12,962.81 ± 1713.19 ^b^	32	0.038
Whole group (kg)	13,298.79 ± 1997.92 ^A^	91	12,315.05 ± 1770.12 ^B^	85	<0.001

Different capital letters denote extremely significant differences between groups (*p* < 0.01), while different lower letters denote significant differences between groups (*p* < 0.05).

**Table 2:** Breeding Index of the Lactating Dairy Cows.

Item	Brush	Control	*p*-Value
Uterine involution rate (%; *n*)	70 (30)	55 (56)	0.105
Luteolysis rate (%; *n*)	100 (30)	95 (56)	0.549
First service conception rate (%; *n*)	35.94 (64)	30.61 (49)	0.694
Days open (d; *n*)	114.38 ± 56.39 (64)	113.80 ± 53.62 (49)	0.956
Number of services per conception (time; *n*)	2.19 ± 1.22 (64)	2.14 ± 1.07 (49)	0.841
30 d-60 d abortion rate (%; *n*)	7.81 (64)	10.20 (49)	0.939
Abortion rate after 60 d (%; *n*)	3.13 (64)	4.08 (49)	0.785

## Data Availability

The data presented in this study are available in the article.
